# A novel cuproptosis-related gene signature predicting overall survival in pediatric neuroblastoma patients

**DOI:** 10.3389/fped.2022.1049858

**Published:** 2022-12-07

**Authors:** Hu Yang, Jun Yang, Hongqiang Bian, Xin Wang

**Affiliations:** Department of General Surgery, Wuhan Children’ Hospital, Tongji Medical College, Huazhong University of Science and Technology, Wuhan, China

**Keywords:** cuproptosis, neuroblastoma, prognosis, tumor microenvironment, model

## Abstract

**Background:**

Cuproptosis is a novel cell death pathway, and the regulatory mechanism in pediatric neuroblastoma (NB) remains to be explored. We amid to investigate cuproptosis-related genes (CRGs) and construct a novel prognostic model for NB.

**Methods:**

To evaluate the role of CRGs on the clinical outcome of pediatric NB, the dataset of pediatric patients with NB of GSE49710 dataset was used to identify CRGs in association with patient overall survival (OS), and TARGET database was used to validate the predictive value of cuproptosis-related signature (CRG-score). The correlation between the CRG-score and the tumor microenvironment (TME), clinicopathological parameters, chemotherapy, and the response to immunotherapy was explored.

**Results:**

Overall, 31 CRGs were associated with OS in the univariate Cox regression analysis. Then, a prognostic model incorporating 9 CRGs was established with the LASSO regression analysis, which could classify all NB patients into two CRG-score groups. The performance of the signature was verified in both internal and external validation cohorts. Multivariate analysis indicated that the CRG-score was an independent prognostic indicator, and stratification analysis still showed a high predictive ability for survival prediction. The CRG-score was associated with age, MYCN status, INSS stage, and COG risk. Additionally, the higher CRG-score group exhibited lower immune scores, immune cell infiltration, and decreased expression of immune checkpoints. Meanwhile, the CRG-score could predict the drug sensitivity of administering chemotherapeutic agents for NB patients.

**Conclusions:**

Our comprehensive analysis of cuproptosis-associated genes in NB provides a new approach for the prediction of clinical outcomes and more effective treatment strategies.

## Introduction

Neuroblastoma (NB) is the most common extracranial solid tumor in infants and children, arising from undifferentiated neural crest cells (1). NB is commonly found in small glands on top of the kidneys and accounts for approximately 7%–10% of all childhood cancers (2, 3). Despite intensive treatment regimens and recent advances, NB still accounts for approximately 15% of childhood cancer-related mortality (2, 4). NB exhibits genetic, clinical, and therapeutic response heterogeneity, limiting the efficacy of current therapeutic strategies (5). Some tumors regress significantly without treatment, in other cases, the disease may appear very aggressive, with recurrence or extensive metastases, be ineffective against aggressive multi-pronged therapy, and ultimately, die of relapse or refractory metastatic disease (1, 5). Clinically, approximately 50% of NB patients have metastatic disease at diagnosis and up to 60% will eventually relapse (4, 6). Through the NB risk classification system from the Children's Oncology Group (COG), the patients were divided into low-, intermediate- and high-risk groups (7). Although low- and intermediate-risk patients generally have favorable outcomes, high-risk patients have a high mortality rate, with a 5-year survival rate of less than 50% despite the recent intensification of treatment (8, 9). The treatment of NB mainly includes a multimodal treatment regimen of chemotherapy, surgery, radiation therapy, and myeloablative chemotherapy followed by stem cell rescue, and immunotherapy (10). The biology and genetic basis of NB are currently being studied, and the combination may be an advantageous therapeutic approach. Therefore, considering the possibility of genetic-related therapy for NB, effective prognostic biomarkers must be explored.

Recently, Tsvetkov et al. (11) found that an abnormal rise of copper ions in human cells may induce cell death with a pathway distinct from known mechanisms of regulated cell death, and copper ions can still induce cell death when blocking known cell death patterns. Their studies have demonstrated that the copper delivered by the copper ionophores to the mitochondria binds directly to the lipified proteins in the cell, forcing them to form long chains and clumps that cause cell death. They also found that copper interferes with iron-sulfur clusters, which are part of several key metabolic enzymes, further reducing the enzyme's activity and pushing the cells into a state of toxic stress that ultimately kills them (11). Thus, this copper-dependent modulated mode of cell death is named cuproptosis. Studies have shown that copper is highly present in the serum or tumor tissue of various cancer patients (12, 13). In addition, copper plays a vital role in the etiology and progression of cancer (14, 15), suggesting that it can serve as a potential biological target for cancer diagnosis or therapy. However, the role of copper ptosis-related genes in NB has not been systematically investigated. Furthermore, accurate models predicting children with NB are lacking.

In this work, a comprehensive analysis of the expression of cuproptosis-related genes (CRGs) was performed in pediatric and young adult patients with NB. Then, we constructed a prognostic model based on CRGs using LASSO regression analysis, which was validated by the GEO cohort. The results of our study indicated that cuproptosis-related signature was an independent prognostic variable for pediatric and young adult patients with NB. Furthermore, the relationships between the signature and the tumor microenvironment (TME) phenotype and drug sensitivity were further examined to identify potential immunotherapy response indicators and novel therapeutic targets.

## Materials and methods

### Data source and collation

The process flow of this work is shown in Supplementary Figure S1. The messenger RNA (mRNA) expression profiles and clinicopathological parameters of 498 NB patients were obtained from the Gene Expression Omnibus (GEO; gene ID: GSE49710) database. Data of 144 NB patients from Therapeutically Applicable Research to Generate Effective Treatments (TARGET) database was obtained as an external validation set. The batch effects of different databases were eliminated by the “ComBat” algorithm in the “sva” R package (16). Additionally, 41 cuproptosis regulators were obtained from the previous publications (11, 17), and are listed in Supplementary Table S1.

### Development and validation of a cuproptosis-related signature

The 41 cuproptosis-related genes (CRG) were further analyzed by univariate Cox regression assessment using the “survival” R package to screen those correlated with the overall survival (OS) of NB. The number of genes was further reduced and gene collinearity was eliminated using Least Absolute Shrinkage and Selection Operator (LASSO) regression analysis *via* the “glmnet” R package and a prognostic model (CRG-score) was established. The regression coefficient of each gene was estimated by applying LASSO Cox regression, and the optimal lambda (*λ*) was derived by 10-fold cross-validation. The CRG-score of all samples in the GEO set was calculated using the following equation based on the expression value of CRGs and the non-zero regression coefficient obtained by LASSO Cox regression analysis.

CRG-score=Exp1∗Coef1+Exp2∗Coef2+Exp3∗Coef3+…+Expi∗Coefi

where the Coefi is the coefficient of each CRG, and Expi is the expression level of each CRG. The CRG-score for each patient in the TARGET set was calculated with the same coefficients and normalized expression microarray data of CRGs. After that, the samples in the two sets were separately stratified into two groups based on the median CRG-score of the training set. Those samples scoring above the median CRG-score were considered high CRG-score level, otherwise, they were low CRG-score level.

### Evaluation of CRG-score prognostic accuracy

The prediction accuracy of the CRG-score was then internally and externally validated. The time-dependent receiver operating characteristic (ROC) was performed using the “timeROC” R package to assess the accuracy of the CRG-score in both GEO and TARGET sets. The “survival” and “survminer” packages were employed to plot Kaplan-Meier (K-M) survival curves. Principal component analysis (PCA) using the “scatterplot3d” package in R software was utilized to better differentiate NB patients of two CRG-score groups.

### Clinical significance of the CRG-score

To further confirm the clinical role of the CRG score, the correlation between the CRG-score and clinical information was assessed using the “limma” package, which included age, gender, MYCN status, INSS stage, histology, and COG risk. Next, we investigated whether the CRG-score was an independent predictor of OS using univariate and multivariate independent prognostic analyses. Moreover, we further conducted a stratified analysis of patients with different clinical parameters to test the prognostic role of the CRG-score.

### Analysis of tumor microenvironment

The “estimate” R package was used to calculate the immune score, estimate score, and stromal score. We further used the ssGSEA algorithm to calculate the enrichment of 13 immune-related functions and 16 infiltrating immune cells and the “limma”, “ggpubr” and “reshape2” R packages were employed to analyze the differences in these immune cells and pathways between the two CRG-score groups (Supplementary Table S2). Moreover, the distribution of 22 types of immune cells was calculated by the CIBERSORT algorithm (Supplementary Table S3), and the association between them and the expression levels of the CRGs of the risk model was evaluated by Spearman correlation analysis. A *p*-value below 0.05 was defined as statistically significant.

### Prediction of chemotherapy and immunotherapy response for NB

The R package “pRRophetic” was performed to predict response to chemotherapy in NB patients. Differences in half-maximal inhibitory concentrations (IC50) value of 251 drugs (Supplementary Table S4) from the Cancer Genome Project (CGP) 2016 database between the two CRG-score level groups was statistically analysed with the help of Wilcoxon rank-sum test. Additionally, the correlation between 47 immune checkpoints and the CRG-score was calculated, and the box plot was plotted by the “reshape2” and “ggplot2” R packages. The 47 immune checkpoints were listed in Supplementary Table S5.

### Functional enrichment analysis

Differently expressed genes (DEGs) between two CRG-score categories were filtered using the “limma” package (|FoldChange| > 1.5, *p* < 0.05). The GO and KEGG enrichment analyses were performed based on DEGs using “clusterProfiler” R package.

### Statistical analysis

Statistical analysis was conducted by the R software (version 4.2.0). Kaplan-Meier approach was used to generate survival curves for the prognostic analysis, and log-rank test was used to check for differences between groups. The *p*-value was bidirectional, and the adjusted *p*-value < 0.05 was statistically significant.

## Results

### Establishment of cuproptosis-related signature

**Figure 1:**
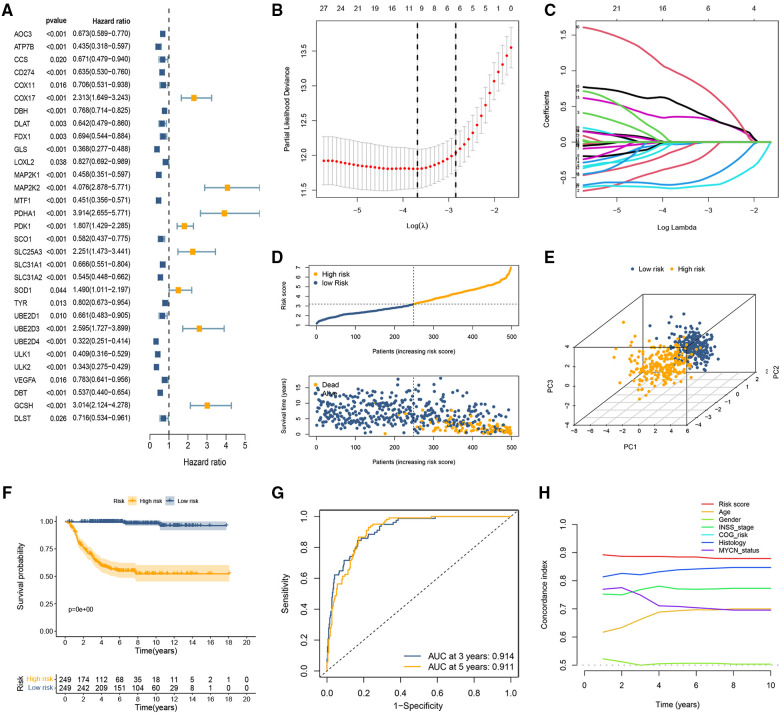
Development of the cuproptosis-related prognostic signature in the training set. (**A**) Forest plot of nine CRGs correlated with OS. (**B,C**) The LASSO Cox regression analysis was performed depending on the optimal *λ* value. (**D**) The distributions of OS status and patient survival status in the training set. (**E**) PCA showed remarkable differences between the two CRG-score groups. (**F**) The Kaplan-Meier curves showed that significant differences were identified for OS between these two risk groups. (**G**) ROC curves evaluating the sensitivity and specificity of the cuproptosis-based prognostic model. (**H**) C-index of the risk score and other clinical parameters.

We performed a univariate Cox regression analysis in the GSE49710 dataset and found that 31 CRGs individually have a possible effect on the patient's OS (*p* < 0.05; Figure 1A). Subsequently, LASSO Cox regression analysis was performed to obtain a prognostic signature. Partial likelihood deviance and LASSO coefficient profiles are shown by vertical dotted line plots, and 9 CRGs (ATP7B, CD274, FDX1, MAP2K1, MAP2K2, PDHA1, UBE2D4, ULK2, and GCSH) were selected to construct a prognostic signature (Figures 1B,C). The CRG-score for each patient in the training set was calculated using the LASSO regression coefficient and the expression level of the 9 CRGs.

CRG-score = (−0.33929 × ATP7Bexp) + (−0.03142 × CD274exp) + (−0.11579 × FDX1exp) + (−0.01715 × MAP2K1exp) + (0.35511 × MAP2K2exp) + (0.54638 × PDHA1exp) + (−0.59282 × UBE2D4exp) + (−0.64734 × ULK2exp) + (0.971007 × GCSHexp)

To classify samples into specific CRG-score level groups, the median risk score was identified as the cutoff point. The samples with CRG-score higher than the threshold were stratified into the high CRG-score level group and the other low CRG-score level group.

### Evaluation of the cuproptosis-related signature

The distribution of CRG-score ordered from low to high. As the CRG-score increased, the death risk for patients gradually increased (Figure 1D). The results of PCA indicated that the CRG-score possessed a good discrimination ability to distinguish between the high- and low-risk subgroups (Figure 1E). The survival probability of the low CRG-score group was evidently higher than the high CRG-score group (Figure 1F). ROC curve indicated that the AUC of the 3- and 5-year OS were 0.914 and 0.911, respectively (Figure 1G). In addition, we compared the signature with previous signatures in NB. ROC curves indicated that our signature achieved significantly favorable predictive power compared with previously published prognostic models (Supplementary Figure S2). We then calculated the C-index of CRG-score and several clinical parameters. The highest C-index of the risk score affirmed the predictive utility of our signature (Figure 1H).

**Figure 2:**
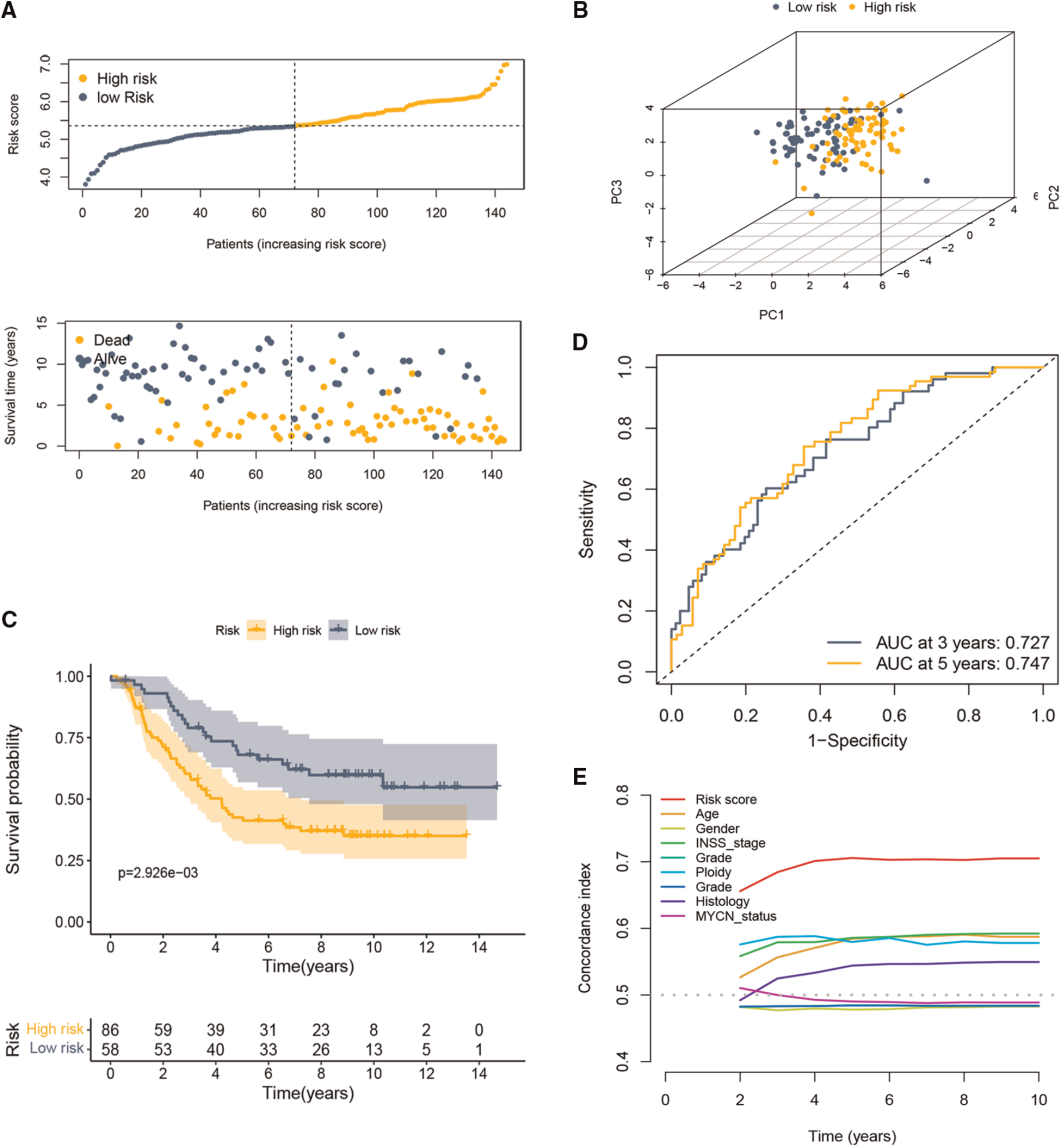
Validation of the cuproptosis-based signature in the testing set. (**A**) The distributions of OS status and patient survival status in the testing set. (**B**) PCA showed that the distribution of patients in two different risk groups is in two directions. (**C**) The Kaplan-Meier curves showed that significant differences were identified for OS between these two risk groups. (**D**) ROC curve at 3 and 5 years. (**E**) C-index of the risk score and other clinical parameters.

The coefficient and formula remained unchanged when evaluating the cases in the TARGET dataset. Based on the same cutoff values in the training set, the patients were stratified into high and low CRG score groups. Consistent with the previous conclusions, patients in the high-risk group had a higher probability of death earlier than those in the low-risk group (Figure 2A). The results of PCA showed that the distribution of patients in two CRG-score groups is in two directions (Figure 2B). Survival analysis revealed that high-risk patients shared significantly worse OS than low-risk patients (Figure 2C). The AUC values for both 3-year and 5-year OS were greater than 0.5 (Figure 2D), indicating the good predictive ability of cuproptosis-based prognostic signature. Likewise, the C-index for the CRG-score was the highest relative to several other clinical parameters (Figure 2E).

### Clinical significance of the cuproptosis-related signature

**Figure 3:**
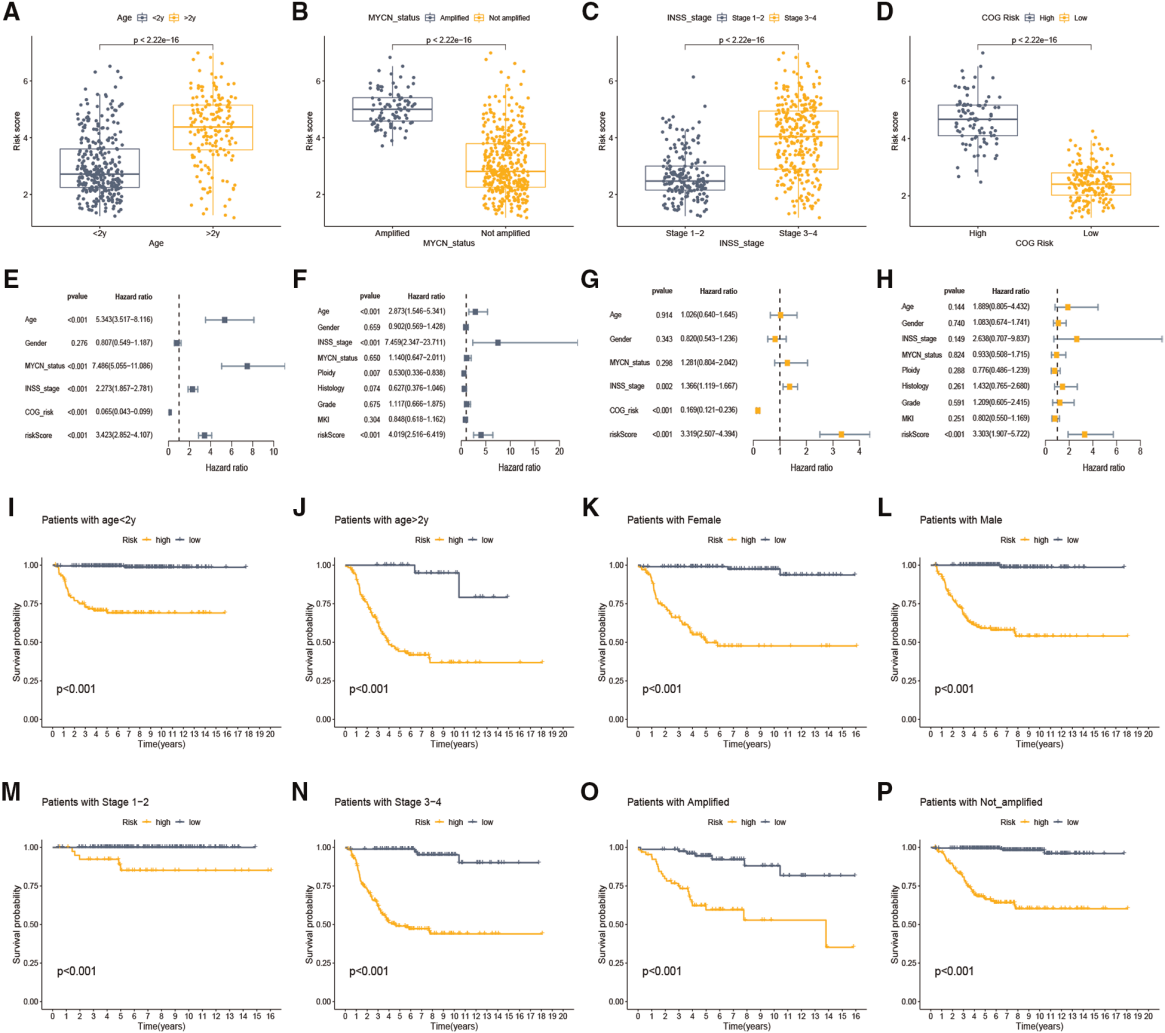
Clinical significance of the cuproptosis-related signature. (**A–D**) The association between clinical traits and CRG-score of NB. (**E,F**) Forest plot of univariable Cox model for survival in the training (**E**) and testing sets (**F**). (**G,H**) Independent prognostic value of CRG-score by multivariate analysis in the training (**G**) and testing sets (**H**). (**I–P**) The prognostic role of the CRG-score was studied in different variable subgroups.

We explored the connection between the CRG-score and clinical traits in the training set and found that the high CRG-score was evidently associated with >2 years old, amplified MYCN status, advanced INSS stage, and high COG risk (Figures 3A–D; all *p* < 0.001). To further determine whether the CRG-score could be used as an independent prognostic indicator, we constructed univariate Cox regression and multivariate Cox regression analyses in training and testing sets. We observed that age, MYCN status, INSS stage, COG risk, and CRG-score in the GEO set were evidently associated with OS, and a summary of the results is displayed in the forest plot (Figure 3E). Similarly, age, INSS stage, ploidy, and CRG-score in the testing set were significantly associated with OS (Figure 3F). Furthermore, we enrolled these factors into a multivariate Cox analysis and found that the CRG-score was an independent prognostic indicator in both sets (Figures 3G,H). Additionally, the prognostic role of the CRG-score was further studied in different variable subgroups. In comparison with high-risk NB patients, low-risk patients manifested a better OS in subclasses layered by age, MYCN status, and INSS stage (all *p* < 0.05, Figures 3I–P).

### Immune cell landscape

**Figure 4:**
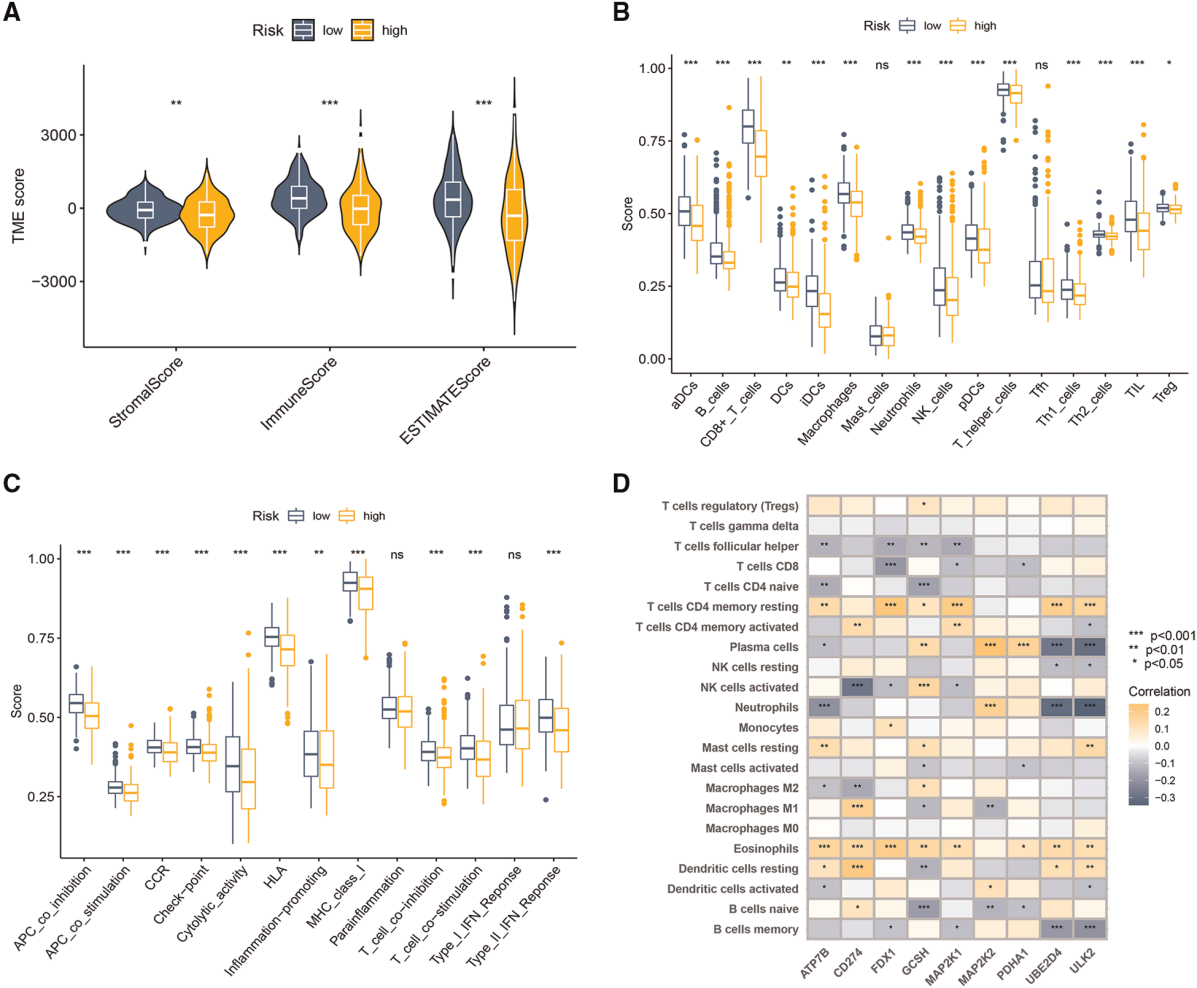
Immune-related analysis between high-risk and low-risk groups. (**A**) The variance analysis of the ESTIMATE score, stromal score, and immune score in the different CRG-score groups. (**B**) The immune infiltration of immune cell types in high and low CRG-score patients. (**C**) Analysis of immune functions in high and low CRG-score patients. (**D**) Correlation analysis of 9 model CRGs and 22 types of immune cells. *p < 0.05, **p < 0.01, ***p < 0.001.

The stromal score, immune score, and ESTIMATE score were all at a much lower level in the high CRG-score sample (Figure 4A). Enrichment scores for most tumor-infiltrating lymphocytes (Figure 4B) and pathways (Figure 4C) were significantly elevated in the low CRG-score group. Further correlation analysis indicated close ties between the expression of 9 model CRGs and different types of infiltrating immune cells (Figure 4D).

### Predictive efficacy of the signature in immunotherapy and chemotherapy

**Figure 5:**
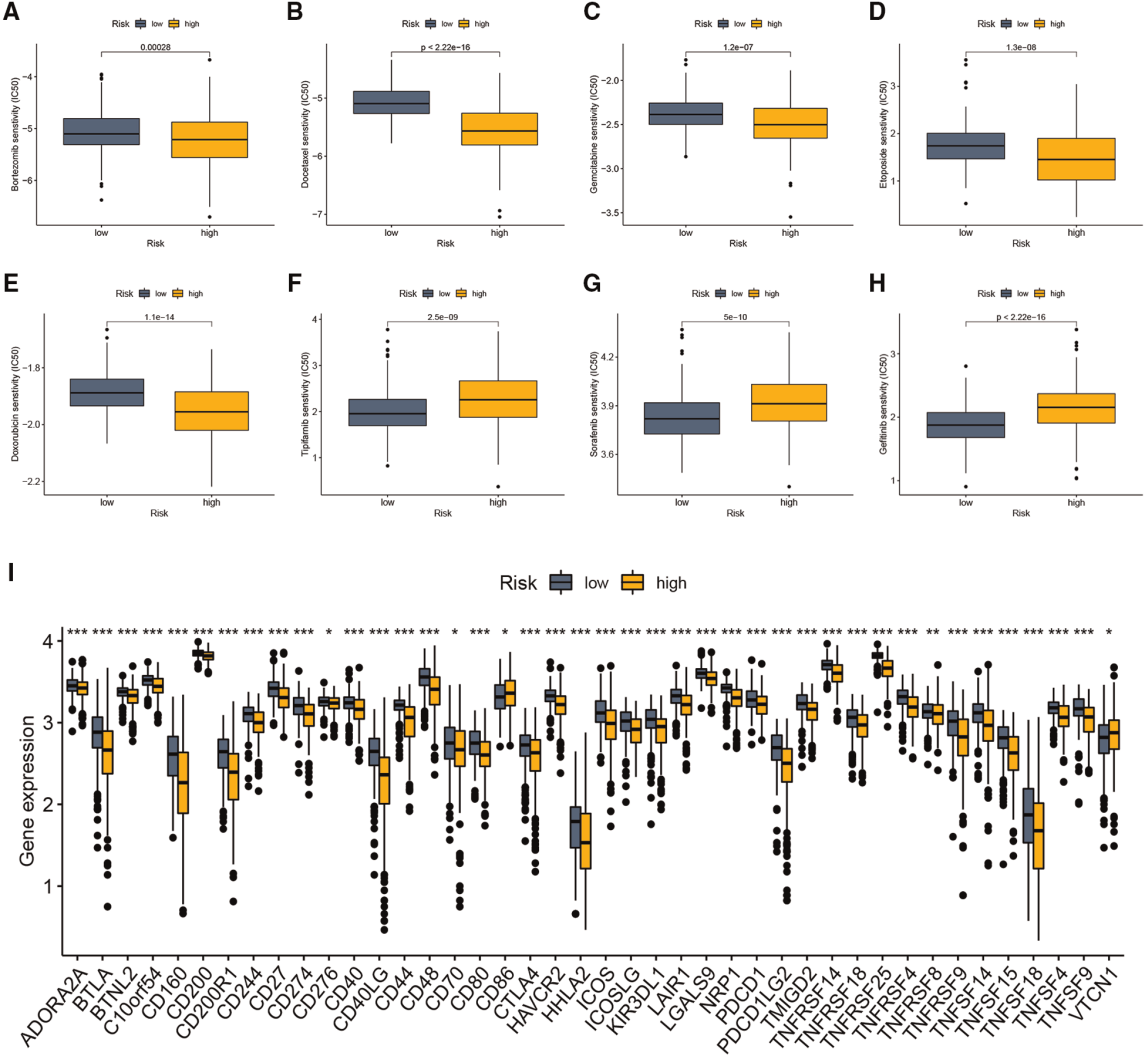
The predictive value of the cuproptosis-related signature in chemotherapy and immunotherapy. (**A–H**) Drug sensitivity analysis between two CRG-score groups. (**I**) The relationship between immune checkpoints and the CRG-score. *p < 0.05, **p < 0.01, ***p < 0.001.

We explored whether this signature could predict the responsiveness of the patient to the chemotherapy and immunotherapy treatment by applying the “pRRophetic” method. As illustrated in Figures 5A–E, the high CRG-score group shows greater sensitivity to Bortezomib, Docetaxel, Gemcitabine, Etoposide, and Doxorubicin compared to the low CRG-score group, and those patients could benefit from the treatment. On the other hand, Tipifarnib, Sorafenib, and Gefitinib could be potentially more effective for the low CRG-score group (Figures 5F,H).

Given the immunophenotype discrepancy of the two CRG-score groups, we further investigated the relationship between the CRG-score and immune checkpoints, including PD-L1 and CTLA4. As illustrated in Figure 5I, the expression of 41 of 47 immune checkpoints was downregulated in the high CRG-score group compared with the low CRG-score group.

### GO and KEGG pathway analysis

**Figure 6:**
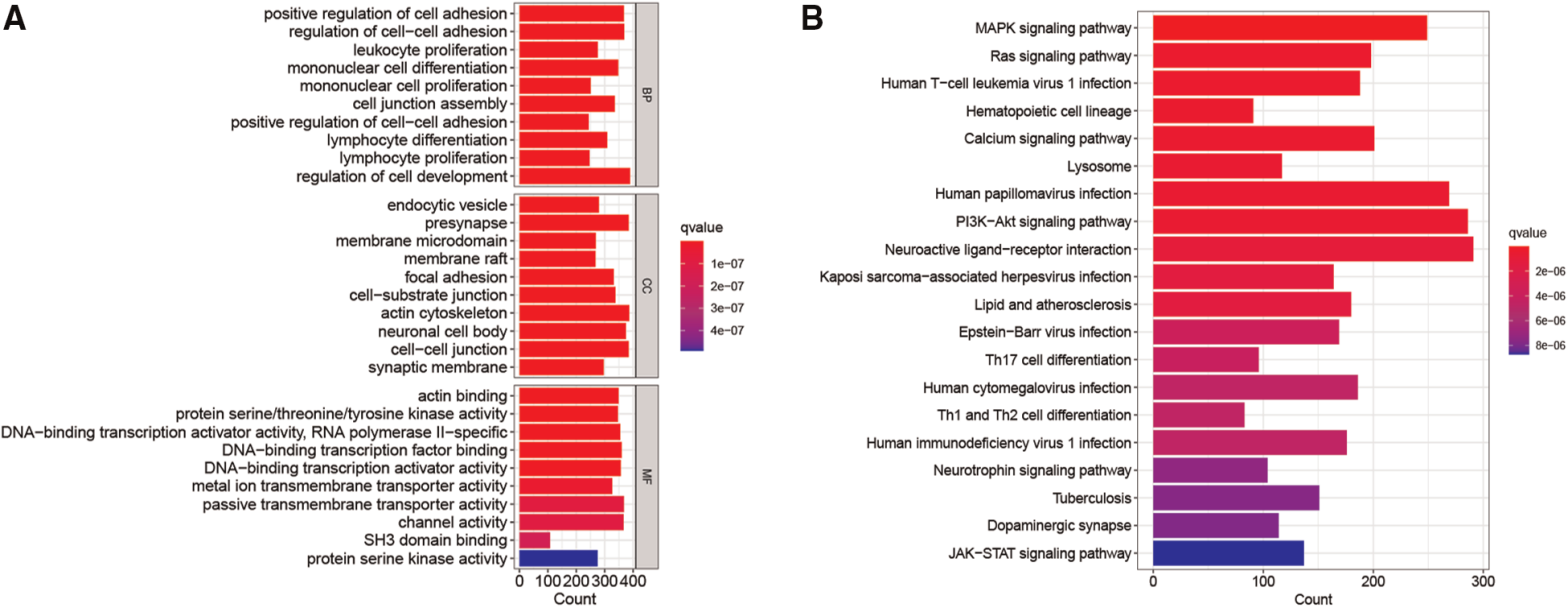
Go and KEGG enrichment analysis of differential genes. (**A**) The bar graph of the GO terms. (**B**) The bar graph of the KEGG pathways.

To further explore the potential biological characteristics of DEGs between the two CRG-score groups, we performed GO and KEGG pathway analysis. The GO analyses demonstrated significant enrichment of positive regulation of cell adhesion, endocytic vesicle, and actin binding (Figure 6A). The KEGG analysis revealed enrichment in several pathways associated with MAPK signaling pathway, Ras signaling pathway, and PI3K-Akt signaling pathway (Figure 6B). These results suggested that cuproptosis-related signature is involved in the initiation and progression of NB.

## Discussion

To explore the relationship between CRGs and the prognosis of NB, we systematically analyzed the transcriptomic data from the GEO and TARGET sets and established a cuproptosis-related signature. The NB patients with high CRG-score had notably shorter OS times than those with low CRG-score in both training and testing sets. The predictive accuracy of the genetic predictor was internally and externally validated. The prognostic signature included 9 CRGs (ATP7B, CD274, FDX1, MAP2K1, MAP2K2, PDHA1, UBE2D4, ULK2, and GCSH). Copper transporter ATP7B is a copper-transporting ATPase that plays a central role in regulating copper homeostasis in the liver, and dysfunctional ATP7B is involved in the development of monogenic Wilson disease (18, 19). Elevated ATP7B expression has been observed in a variety of cancers, and its expression is associated with cancer prognosis and treatment outcome with platinum-based chemotherapy (20, 21). Programmed death ligand 1 (PD-L1, CD274) is a major co-suppressive checkpoint signal regulating immune escape in cancer patients (22). Various types of cancer express high levels of PD-L1 and suppress antitumor immunity by interacting with the PD-1 receptor of T lymphocytes (23, 24). Therefore, blocking the PD-L1/PD-1 pathway is recognized as the gold standard for the development of new immune checkpoint blockade (ICB) and combination therapies (22). Several studies have identified the diagnostic and prognostic potential of PD-1/PD-L1 in neuroblastoma (25, 26). Zuo et al. (27) revealed that 11 of 31 (35%) NB patients expressed PD-L1 and high PD-L1 expression was associated with worse OS. The FDX1 gene is a small iron-sulfur protein involved in the metabolism of steroids, bile acids, and vitamin D (28). Recently, FDX1 has been shown to be a key regulator of copper-induced death that can positively regulate specific metabolic pathways of cuproptosis (11).

The immune landscape, as the major component of the TME, determines tumorigenesis and disease progression. Immune cells are critical components of this TME, and they can influence the ability of tumor cells to proliferate and respond to treatment (29, 30). Many studies have also suggested a strong link between copper metabolism and tumor immunity (31, 32). Voli et al. (32) revealed that intratumoral opper promotes PD-L1 expression at the mRNA and protein levels in cancer cells, and RNA-sequencing revealed that copper regulates a key signaling pathway that mediates PD-L1-driven cancer immune evasion. Conversely, copper chelators promoted ubiquitin-mediated PD-L1 degradation and increased the number of CD8+ T cells and natural killer cells. Although, no definitive reports have indicated a direct relationship between cuproptosis and immune cell infiltration in NB. In this study, infiltrating immune cells were explored between two CRG-score subgroups. The results of the comparative immune microenvironment analysis indicate that most infiltrating immune cells were less infiltrated in the high-risk group, such as Dendritic cells, B cells, CD8+ T cells, and NK cells. With their potent antigen-presenting ability, dendritic cells have long been considered a key component of antitumor immunity. Activated dendritic cells are key to the development of long-term and effective anticancer immunity (33, 34). As an important part of the TME, B cells can directly produce antibodies or present tumor-associated antigens to T cells, killing tumor cells or increasing T-cell antigen presentation, thereby inducing tumor regression (35). CD8+ T cells play a key role in eliminating malignant cells and are the backbone of current successful cancer immunotherapies (36). Tumor-infiltrating CD8+ T cells were correlated with favorable clinical outcomes in patients with advanced cancers (37). In this study, we compared the difference in TME scores between the two groups and found that the high CRG-score group had lower TME scores. Additionally, most immune-related pathways were inactive in the high CRG-score group. These data indicated the high CRG-score group demonstrating immunosuppressive phenotype.

Immunotherapy has made great progress in the treatment of cancer in recent years, and it has been found that a promising approach to achieve immunity against cancer is to block the immune checkpoint pathway (38–40). Immunotherapy holds great promise for treating pediatric cancer. The anti-GD2 antibody Dinutuximab was recently shown to significantly improve the prognosis of NB patients (41). However, NB exhibits low immunogenicity due to their low tumor mutational burden and lack of MHC-I expression, resulting in most patients benefiting from treatment with immune checkpoint inhibitors (ICIs), and the 5-year survival rate of high-risk NB patients remains below 50% (42, 43). Therefore, screening for sensitive predictors of immunotherapy is important for clinical treatment. In this study, we investigated the relationship between the CRG-score and the expression of immune checkpoint-related genes. We observed elevated expression levels of most immune checkpoints, including PD-1 and PD-L1, in the low-risk group, implying that these patients could benefit from ICIs. In addition to immunotherapy, the cuproptosis-related signature was also associated with high sensitivity to chemotherapy and targeted agents. The correlation results between the CRG-score and drug sensitivity suggested that NB patients with high CRG-score may benefit from chemotherapy with Gemcitabine, Etoposide, and Doxorubicin, while low CRG-score patients may benefit from Tipifarnib, Sorafenib, and Gefitinib. Taken together, the cuproptosis-related signature is expected to be used to predict response to immunotherapy and targeted therapy and also provide new ideas for the selection of reasonable chemotherapy and targeted agents.

There were some shortcomings in our study. First, the number of NB cases in the TARGET database was small. Second, the expression levels of all 9 CRGs in clinical samples and cell lines should be detected by qRT-PCR or Western blot. Finally, the exact mechanism of the 9 CRGs in NB needs to be further investigated *in vivo* and *in vitro*.

## Conclusions

The present study constructed a cuproptosis-related signature to predict the prognosis and immune characteristics of NB patients. It may serve as a novel biomarker for prognostic assessment and individualized treatment of NB patients.

## Data Availability

Publicly available datasets were analyzed in this study. This data can be found here: The datasets used in this study are publicly available, which can be found in GEO (gene ID: GSE49710; https://www.ncbi.nlm.nih.gov/geo/) and TARGET databases (https://ocg.cancer.gov/programs/target/data-matrix).
