# Supplementary figures and images for: A novel cuproptosis-related gene signature predicting overall survival in pediatric neuroblastoma patients
Source: Front Pediatr. 2022 Dec 7;10:1049858. doi: 10.3389/fped.2022.1049858 (PMC9768227; doi:10.3389/fped.2022.1049858)

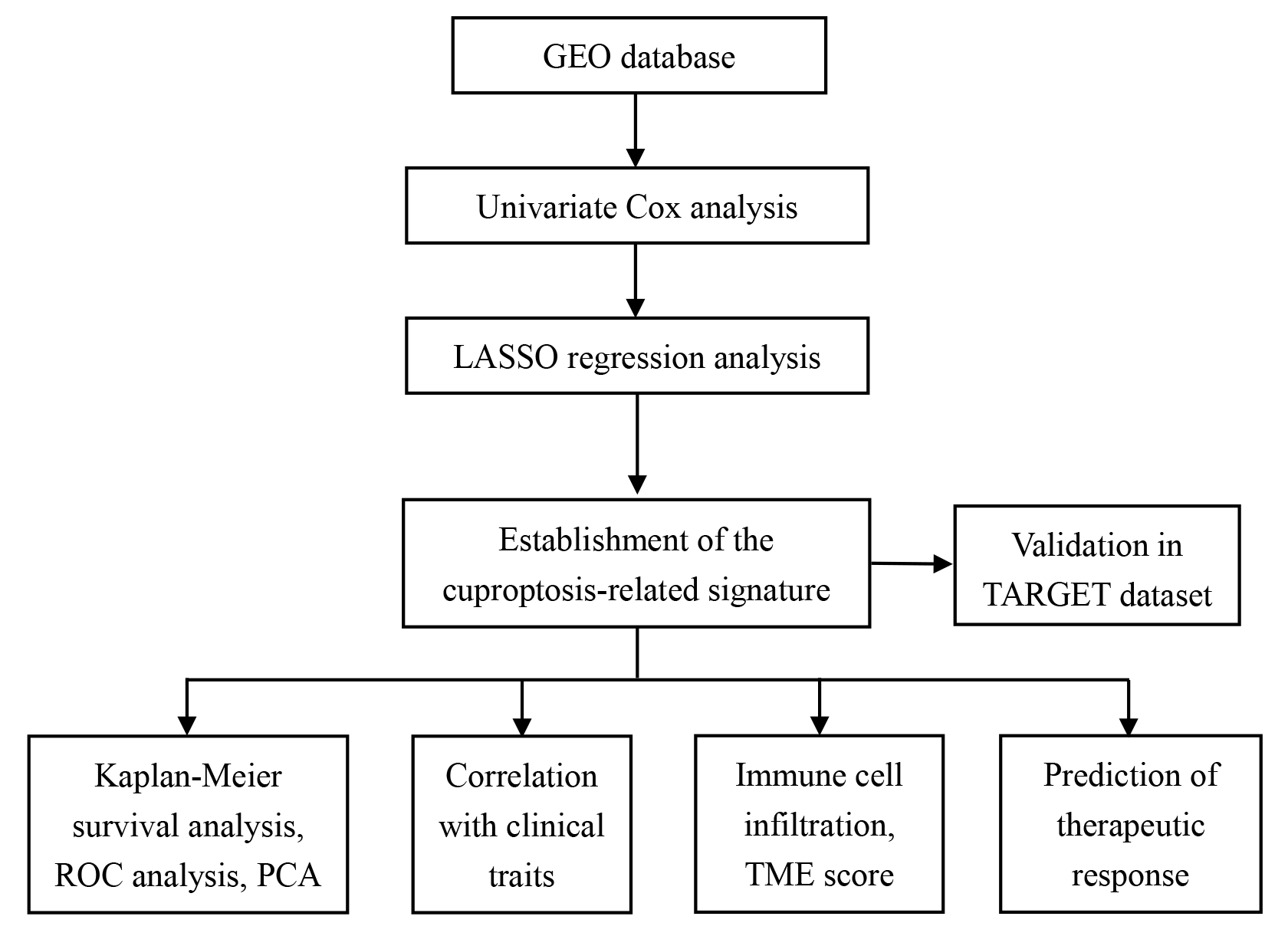

Supplement: Supplementary file 2 [file Image1.tif]

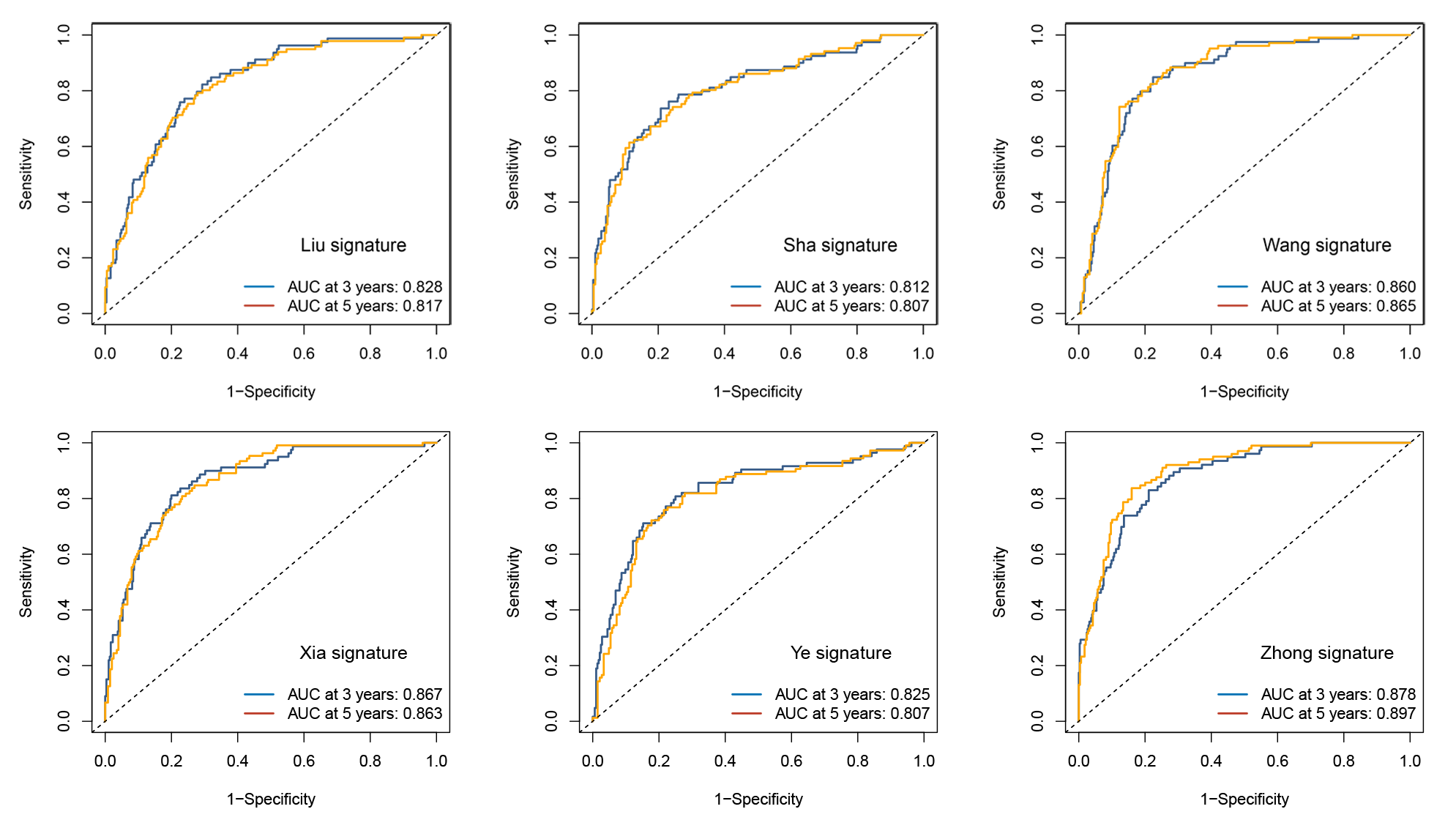

Supplement: Supplementary file 3 [file Image2.tif]
